# Diagnostic Accuracy of 4D CT in Detecting Parathyroid Adenoma Compared With Ultrasound and Sestamibi SPECT/CT in Primary Hyperparathyroidism: A Retrospective Study

**DOI:** 10.7759/cureus.89110

**Published:** 2025-07-31

**Authors:** Somdatta Giri, Krishnan Nagarajan, Balamourougan Krishnaraj, Debasis Gochhait, Madhusudhanan Ponnusamy, Anitha Sebastian, Baskararaj Sriviruthi, Jayaprakash Sahoo, Sadishkumar Kamalanathan, Dukhabandhu Naik

**Affiliations:** 1 Endocrinology, Jawaharlal Institute of Postgraduate Medical Education and Research, Puducherry, IND; 2 Endocrinology and Metabolism, All India Institute of Medical Sciences, Kalyani, Kalyani, IND; 3 Radiodiagnosis, Jawaharlal Institute of Postgraduate Medical Education and Research, Puducherry, IND; 4 Surgery, Jawaharlal Institute of Postgraduate Medical Education and Research, Puducherry, IND; 5 Pathology, Jawaharlal Institute of Postgraduate Medical Education and Research, Puducherry, IND; 6 Nuclear Medicine, Jawaharlal Institute of Postgraduate Medical Education and Research, Puducherry, IND

**Keywords:** diagnostic accuracy, four-dimensional computed tomography (4d ct), hyperparathyroidism primary, parathyroid gland adenoma, percentage arterial enhancement, spect/ct, ultrasound (usg)

## Abstract

Background

The accurate preoperative localization of parathyroid adenomas is crucial for minimally invasive parathyroidectomy (MIP) in primary hyperparathyroidism (PHPT). This study assessed the diagnostic performance of four-dimensional computed tomography (4D CT) in detecting parathyroid adenomas, compared with ultrasound (USG) and technetium methoxy isobutyl isonitrile single photon emission computed tomography (99mTc-sestamibi SPECT/CT).

Methods

We retrospectively analyzed 53 patients with biochemically confirmed PHPT who underwent all three preoperative imaging modalities, followed by parathyroidectomy from January 2020 to January 2025. Imaging findings were validated against intraoperative localization, histopathology, and intraoperative parathyroid hormone (PTH) dynamics. Multi-gland diseases were excluded. Sensitivity, positive predictive value (PPV), and concordance were calculated. Percentage arterial enhancement (PAE) was analyzed as a radiological marker.

Results

The mean age was 42.7 ± 14.7 years, with 29 (54.7%) women. Forty-nine (88.7%) patients had typical adenomas, three (0.05%) had carcinoma, and one (0.01%) had an atypical adenoma. The majority of lesions (24, 45%) were located in the right inferior parathyroid gland, followed by the left inferior (16, 30%). Overall, preoperative imaging was able to localize 50/53 (94.3%) lesions correctly. 4D CT correctly localized 45 lesions, outperforming USG and 99mTc-sestamibi SPECT/CT by identifying eight and 12 additional lesions, respectively. Sensitivity was highest for 4D CT (88.2%), followed by 99mTc-sestamibi SPECT/CT (82.4%) and USG (72.6%), with all three modalities showing high PPV (>94%). Among small adenomas (<20 mm), 4D CT demonstrated superior detection (21/21, 100%) compared to USG (16/21, 76.2%) and 99mTc-sestamibi SPECT/CT (15/21, 71.4%). Dynamic enhancement patterns on 4D CT distinguished adenomas from mimickers. However, applying a fixed PAE cutoff (128.9%), as previously proposed, yielded limited sensitivity (75%) and specificity (31.6%).

Conclusion

4D CT outperformed USG and 99mTc-sestamibi SPECT/CT in localizing parathyroid adenomas in PHPT and was particularly useful when USG or 99mTc-sestamibi SPECT/CT results were inconclusive. While all three modalities showed high positive predictive value, 4D CT localized additional lesions missed by others. Its dynamic contrast patterns effectively differentiated adenomas from mimics. However, the utility of a fixed PAE cutoff was limited by protocol-dependent variability, indicating a need for tailored thresholds.

## Introduction

Primary hyperparathyroidism (PHPT) is the most common cause of hypercalcemia and is predominantly caused by a solitary parathyroid adenoma in 80%-90% of cases [1]. It is characterized by the excessive secretion of parathyroid hormone (PTH). PHPT leads to disturbances in calcium homeostasis and bone metabolism [2]. The only definitive treatment is the surgical resection of the affected gland [3]. In recent years, minimally invasive parathyroidectomy (MIP) has largely replaced traditional bilateral neck exploration, especially in cases of solitary adenoma [4-6]. This shift in surgical approach underscores the importance of the accurate preoperative localization of the adenoma, which determines the feasibility and success of targeted surgical intervention.

Several imaging modalities are available for preoperative localization. Ultrasound (USG) is widely accessible, cost-effective, and free of ionizing radiation; however, its sensitivity is highly operator-dependent and may range from 55% to 87% [7]. Its limitations are most evident when adenomas are located in ectopic sites such as the retrotracheoesophageal regions or mediastinum. Technetium methoxy isobutyl isonitrile single photon emission computed tomography (99mTc-sestamibi SPECT/CT) offers a broader imaging field and can detect ectopic glands, with reported sensitivity ranging from 60% to 90% [8-10]. However, its performance diminishes significantly in multiglandular disease, parathyroid hyperplasia, or small adenoma. Moreover, false positives may arise from uptake in thyroid nodules or lymphadenopathy due to sestamibi’s nonspecific affinity for metabolically active tissues [11].

Four-dimensional computed tomography (4D CT), first used for parathyroid imaging in 2006, has emerged as a highly promising tool [12]. It combines anatomical detail with dynamic contrast enhancement across three phases, unenhanced, arterial, and venous, allowing the identification of parathyroid adenomas based on enhancement characteristics and vascular anatomy. Initially used in reoperative or inconclusive cases, 4D CT is now gaining ground as a frontline imaging modality due to its superior spatial resolution and ability to detect ectopic or small adenomas. Recent literature has highlighted the potential of percentage arterial enhancement (PAE) as an objective radiological marker, calculable from just two phases, baseline and arterial. Goroshi et al. demonstrated high diagnostic accuracy in distinguishing parathyroid lesions from thyroid tissue and lymph nodes with a PAE cutoff of 128.9% [13]. If validated through further studies, this dual-phase protocol may offer a safer alternative to the full four-phase protocol, thus reducing the radiation exposure. Building on these insights, the present study was designed to evaluate the diagnostic accuracy of 4D CT in PHPT and to further delineate the radiological characteristics of parathyroid adenomas, including the potential application of PAE.

## Materials and methods

Study design and setting

This retrospective study was conducted in the department of endocrinology at an academic research institute in India between January 2020 and January 2025. Electronic medical records from the departmental registry of PHPT patients were reviewed for demographic, clinical, and biochemical data. USG, 99mTc-sestamibi SPECT/CT, and 4D CT images were retrieved from the hospital picture archiving and communication system (PACS). This study was performed in line with the principles of the Declaration of Helsinki after obtaining approval from the Institutional Ethics Committee for Observational Studies of the Jawaharlal Institute of Postgraduate Medical Education and Research (ethics number: JIP/IEC-O5120241445). Consent waiver was granted, considering the retrospective nature of the study.

Study population

Eligibility Criteria

Patients were eligible for inclusion if they had (i) a biochemical diagnosis of PHPT, defined by a serum calcium level above the upper limit of normal (>10.5 mg/dL), along with an inappropriately elevated or unsuppressed intact parathyroid hormone (iPTH) level (>30 pg/mL); (ii) all three preoperative imaging studies (4D CT, USG, and 99mTc-sestamibi SPECT/CT) for parathyroid localization; and (iii) subsequent parathyroidectomy. Patients were excluded if they had (i) secondary or tertiary hyperparathyroidism and (ii) multi-gland involvement as occurs in syndromic etiologies. Subjects with incomplete medical or imaging records were also excluded.

Data Collection

Demographic data, including age, gender, and biochemical parameters (calcium, phosphorus, albumin, 25-hydroxyvitamin D {25(OH)D}, and iPTH), were extracted from electronic medical records. Imaging data from USG, 99mTc-sestamibi SPECT/CT, and 4D CT reports were reviewed. Imaging characteristics included the site and size of the lesion.

Imaging Protocol

All 4D CT scans were performed using a 64-detector (128-slice) Somatom Definition scanner (Siemens, Erlangen, Germany). After the baseline imaging, arterial-phase imaging was taken at a 10-15-second delay, followed by venous- and delayed-phase acquisitions at 60-second intervals. Scanning parameters included 120 kVp, 220 mAs, 2 mm slice thickness, and a 0.5-second rotation time. Lesions were assessed for enhancement characteristics expressed in Hounsfield units (HU). These features were also compared with those of thyroid parenchyma and nearby cervical lymph nodes to aid in differential diagnosis. Percentage arterial enhancement (PAE) was calculated by subtracting the HU in the basal phase from those in the arterial phase and dividing the result by the basal phase HU ({(HU in arterial phase-HU in basal phase)/HU in basal phase}×100). The primary objective of this study was to evaluate the diagnostic accuracy of 4D CT in detecting parathyroid adenomas compared to USG and 99mTc-sestamibi SPECT/CT in patients with PHPT. The secondary objectives were to compare the enhancement pattern of parathyroid lesions with that of thyroid parenchyma and nearby lymph nodes. Ultrasound was performed using a Siemens S3000 system with a 9 MHz linear probe, with the patient in the supine position and neck extended. Grayscale and Doppler features of the parathyroid glands were assessed, including number, location, size, morphology, echotexture, and vascularity. For 99mTc-sestamibi SPECT/CT, 16 mCi of 99mTc-sestamibi was injected intravenously into the right cubital fossa. Static anterior images of the neck and mediastinum were acquired 10 minutes post-injection (128×128 matrix), followed by SPECT/CT imaging of the head and mediastinum. Delayed static images were obtained at 1.5 hours post-injection. Imaging was performed using a Siemens Symbia T6 dual-head system, with a radiopharmaceutical purity of 99.6%.

Reference Standard

The reference standard for the localization of parathyroid adenomas was the intraoperative identification of the pathological gland by the surgeon. Accordingly, surgical notes were carefully reviewed to document the intraoperative findings. In cases where all three imaging modalities (USG, 99mTc-sestamibi SPECT/CT, and 4D CT) indicated the same anatomical site, this was considered concordant imaging, and the surgeon proceeded with targeted excision based on the preoperative localization. The intraoperative intact parathyroid hormone (iPTH) measurements were then analyzed; a drop of more than 50% from the pre-incision value, measured 10 minutes after gland excision, was considered indicative of successful removal, in line with the Miami criterion [14]. In cases where there was no significant PTH drop but the excised tissue was histopathologically confirmed to be an adenoma, the localization was still considered accurate, recognizing that incomplete gland removal or parathyroid tissue seeding may sometimes prevent a complete biochemical response. If histopathology was not available, the intraoperative PTH trend was relied upon solely to assess adequacy. When the biopsy described the excised tissue as parathyroid in origin but was inconclusive regarding adenoma, yet there was a significant intraoperative PTH decline, the localization was again deemed correct. In scenarios where there was discordance among the three imaging modalities, the surgical notes were reviewed to determine which gland had been removed. If either the histopathological diagnosis confirmed an adenoma or a significant intraoperative PTH drop was observed, that particular gland was accepted as the correct source. If the surgeon’s decision was found to be incorrect, then a second surgery was performed, and the findings of the second surgery were noted accordingly.

Measures for Diagnostic Accuracy

A true positive result for an imaging modality was defined as the correct localization of the parathyroid adenoma, as confirmed by intraoperative findings, PTH dynamics, and histopathology as described previously. If an imaging modality failed to detect any lesion in a biochemically confirmed case of PHPT, it was considered a false negative. When an imaging modality localized a lesion to an incorrect site, one that was ultimately not found to harbor the pathology, it was categorized as a false positive. As the study exclusively included patients with biochemically proven PHPT and did not involve individuals with secondary hyperparathyroidism or healthy subjects as controls, there were no true negatives. Consequently, the sensitivity, positive predictive value (PPV), and diagnostic accuracy of each imaging modality were calculated.

Biochemical analysis

Utilizing a Beckman Coulter analyzer, biochemical parameters such as creatinine, albumin, calcium, and phosphorus were analyzed. In our laboratory, the normal range for serum calcium and phosphorus was 8.8-10.2 mg/dL and 2.5-4.5 mg/dL, respectively. Direct chemiluminometric technology (ADVIA Centaur XP PTH, Siemens, Erlangen, Germany), a two-site sandwich immunoassay, was used to quantify plasma iPTH. The detection range is 0.488-233 pmol/L or 4.6-2200 pg/mL. For the assay, the normal range for plasma iPTH is 18.4-80.1 pg/mL (1.95-8.49 pmol/L). This assay has a within-run coefficient of variation (CV) of 8.0% and a within-laboratory (total) CV of 10.0%. The ADVIA Centaur 25(OH)D assay, an 18-minute, one-pass antibody competitive immunoassay, was used to quantify serum 25(OH)D. It can detect between 4.2 and 150 ng/mL (10.5 and 375 nmol/L). For the assay, the normal range for serum vitamin D is 20-100 ng/mL. This assay’s within-run and total CV were 7.0% and 11.1%, respectively.

Statistical analysis

Statistical analyses were performed using SPSS version 19.0 for Windows (IBM Corp., Armonk, NY) to evaluate the diagnostic performance of each imaging modality. Categorical variables, such as gender and lesion location, were expressed as frequencies and percentages. Continuous variables, including lesion size and biochemical parameters, were summarized as mean±standard deviation (SD) or median with interquartile range (IQR), depending on data distribution. Sensitivity and PPV with 95% confidence interval (95% CI) of 4D CT, USG, and 99mTc-sestamibi SPECT/CT were calculated based on the established reference standard. The McNemar test was employed to compare the sensitivities of the modalities in the same cohort of patients. Attenuation values in HU in CT scan for parathyroid lesions, thyroid parenchyma, and lymph nodes were compared by ANOVA. Concordance between any two imaging modalities was assessed through kappa statistics. All statistical tests were two-sided, and a p-value of <0.05 was considered statistically significant.

## Results

A total of 53 patients diagnosed with primary hyperparathyroidism were included in the study. The mean age of the participants was 42.7±14.7 years, with a female predominance (29, 54.7%). The average lesion size was 21.1±9.7 mm. Median baseline iPTH level was 328 pg/mL (198.5-726.6), and mean serum calcium was 11.7±1.0 mg/dL.

Diagnostic performance of imaging modalities

We included 53 PHPT patients with single gland disease, where all three imaging modalities were utilized. Among these patients, 49 had typical adenomas, three had carcinoma, and one had an atypical adenoma. The majority of lesions (24, 45%) were located in the right inferior parathyroid gland, followed by the left inferior (16, 30%), left superior (seven, 13%), and right superior (three, 5%) positions (Table 1).

**Table 1:** Baseline Demographic and Biochemical Parameters of the Study Population. *Values are presented as mean±SD or median (interquartile range). ^#^Locations identified in three cases by neck exploration. ^$^The three ectopic locations were one in the left tracheoesophageal groove, one in the right tracheoesophageal groove, and one in the suprasternal location. SD, standard deviation; PHPT, primary hyperparathyroidism; 25(OH)D, 25-hydroxyvitamin D; iPTH, intact parathyroid hormone

Parameter	Value
Age (years)*	42.7±14.7
Female, n (%)	29 (54.7)
iPTH (pg/mL)*	328 (198.5-726.6)
25(OH)D (ng/mL)*	19.0 (12.7-25.6)
Calcium (mg/dL)*	11.7±1.0
Phosphate (mg/dL)*	2.5±0.8
Urine calcium/creatinine ratio	0.48 (0.27-0.69)
Lesion size (mm)*	22.39±10.6
Total PHPT cases	53
Typical adenoma, n (%)	47 (88.68)
Atypical adenoma, n (%)	1 (0.01)
Carcinoma, n (%)	3 (0.05)
Location identified by imaging,^#^ n (%)	50 (94.34)
Right inferior, n (%)	24 (45.28)
Left inferior, n (%)	16 (30.19)
Left superior, n (%)	7 (13.21)
Right superior, n (%)	3 (5.66)
Ectopic,^$^ n (%)	3 (5.66)

**Figure 1:**
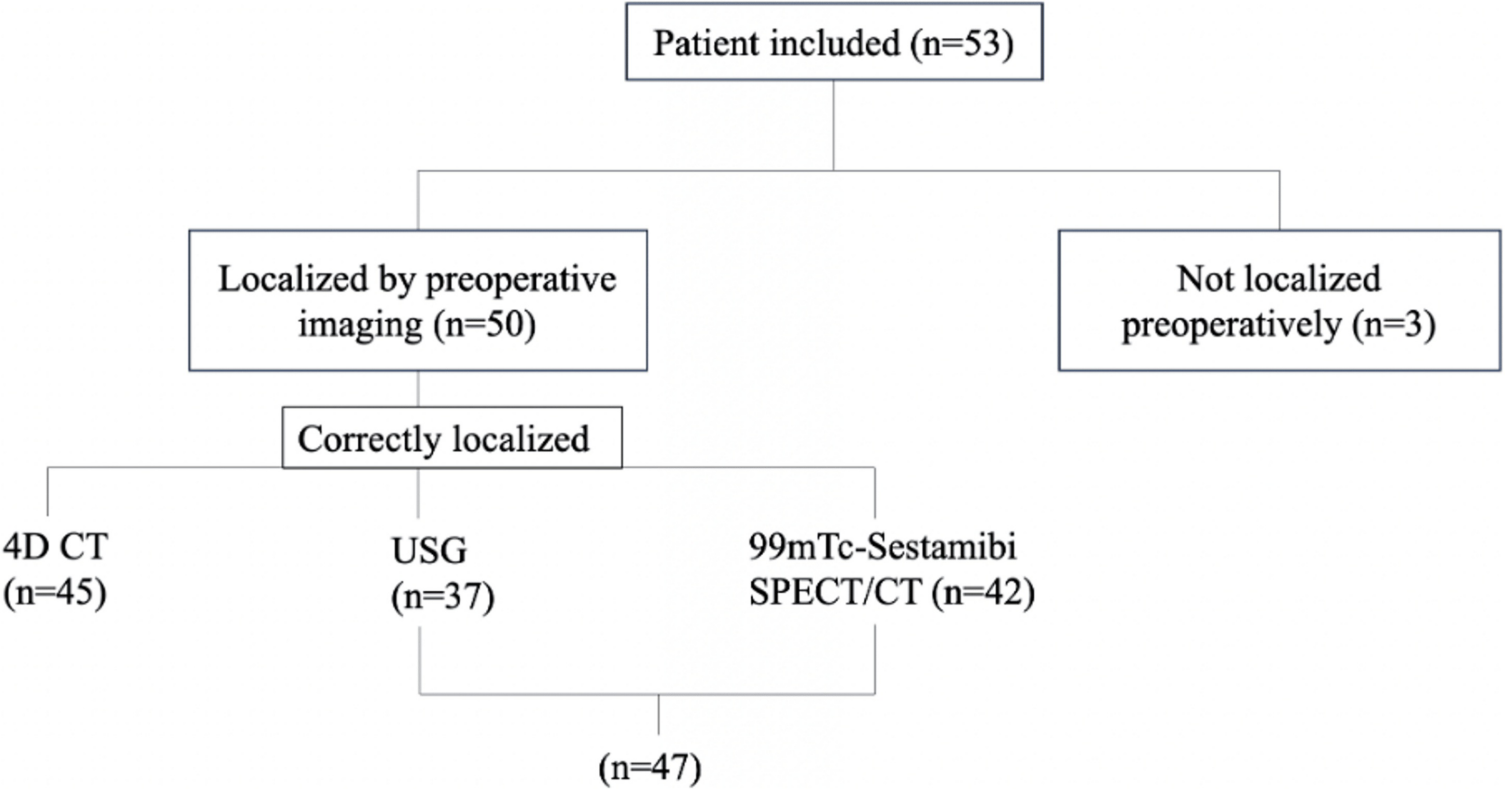
Flow Diagram Showing Preoperative Localization by Imaging. 4D CT, four-dimensional computed tomography; USG, ultrasound; 99mTC-sestamibi SPECT/CT, technetium methoxy isobutyl isonitrile single photon emission computed tomography

4D CT correctly identified 45 lesions (true positives) and yielded two false positives, one of which was correctly localized by both USG and 99mTc-sestamibi SPECT/CT, while the other remained undetected by all three modalities (Figure 1). Additionally, 4D CT failed to detect six lesions (false negatives), of which three were identified by USG, and two additional lesions were localized when 99mTc-sestamibi SPECT/CT was used in combination (Table 2). A detailed description of the eight lesions missed by CT is provided in Table 3. Notably, 4D CT was able to detect eight lesions missed by 99mTc-sestamibi SPECT/CT and 12 lesions missed by USG, suggesting its added value, especially in cases with inconclusive or negative findings on other modalities. Kappa statistics showed slight agreement between modalities: 4D CT versus USG (κ=0.165) and 4D CT versus 99mTc-sestamibi SPECT/CT (κ=0.171), indicating that their localizing abilities are not strongly concordant. The sensitivity, PPV, and overall accuracy of each modality are summarized in Table 4. To further evaluate performance, lesions were stratified by size using a 20 mm cutoff. Among the 21 small adenomas, 4D CT correctly identified all cases (21, 100%), whereas USG and 99mTc-sestamibi SPECT/CT detected 16 (76.2%) (p=0.06) and 15 (71.4%) (p=0.03), respectively.

**Table 2:** Diagnostic Performance of Imaging Modalities. 4D CT, four-dimensional computed tomography; USG, ultrasound; 99mTC-sestamibi SPECT/CT, technetium methoxy isobutyl isonitrile single photon emission computed tomography

	USG	99mTc-sestamibi SPECT/CT	4D CT
True positive	37	42	45
False positive	2	2	2
False negative	14	9	6

**Table 3:** Description of Lesions That Are Missed in 4D CT Scan. ✔: Lesion correctly localized by the imaging modality. X: Lesion incorrectly localized by the imaging modality. –: Lesion missed by the imaging modality. M, male; F, female; iPTH, intact parathyroid hormone; LIPA, left inferior parathyroid adenoma; LSPA, left superior parathyroid adenoma; RIPA, right inferior parathyroid adenoma; RSPA, left superior parathyroid adenoma; 4D CT, four-dimensional computed tomography; USG, ultrasound; 99mTC-sestamibi SPECT/CT, technetium methoxy isobutyl isonitrile single photon emission computed tomography

4D CT	USG	99mTc-sestamibi SPECT/CT	Age and sex	Location	iPTH (pg/mL)	Size (mm)
X	✔	✔	49 years, M	LSPA	420	27
X	–	–	77 years, F	LSPA	282	23
–	X	–	35 years, F	LIPA	125	-
–	–	✔	55 years, M	LSPA	124	21
–	✔	✔	65 years, F	RIPA	294	-
–	–	✔	47 years, F	LIPA	1866	-
–	✔	X	42 years, F	RIPA	1899	35
–	✔	✔	25 years, F	RIPA	2000	35

**Table 4:** The Sensitivity, PPV, and Accuracy Values of the Imaging Modalities. The McNemar p-value of USG versus 99mTc-sestamibi SPECT/CT, 0.302; USG versus CT, 0.07; and 99mTc-sestamibi SPECT/CT versus CT, 0.581. 4D CT, four-dimensional computed tomography; USG, ultrasound; 99mTC-sestamibi SPECT/CT, technetium methoxy isobutyl isonitrile single photon emission computed tomography; PPV, positive predictive value; CI, confidence interval

	USG	99mTc-sestamibi SPECT/CT	4D CT
Sensitivity, % (95% CI)	72.5 (58.2-84.1)	82.3 (69.1-91.6)	88.2 (76.1-95.5)
PPV, % (95% CI)	94.8 (93.9-95.6)	95.4 (94.8-95.9)	95.7 (95.3-96.1)
Accuracy, % (95% CI)	69.8 (55.6-81.6)	79.2 (65.8-89.1)	84.9 (72.4-93.2)

Overall, preoperative imaging successfully localized the parathyroid lesion in 50 out of 53 patients (94.3%). In one case, only USG identified a lesion; however, there was no intraoperative iPTH drop, and histopathological confirmation was unavailable. In a second case, both 4D CT and USG localized lesions at different sites, but neither corresponded to the actual lesion. In the third case, 4D CT alone suggested an incorrect localization. All three patients eventually underwent a second surgery with bilateral neck exploration, which successfully identified the true lesions: the right superior parathyroid gland in the first two cases and the left superior in the third.

Radiological features of parathyroid adenoma on 4D CT

**Figure 2:**
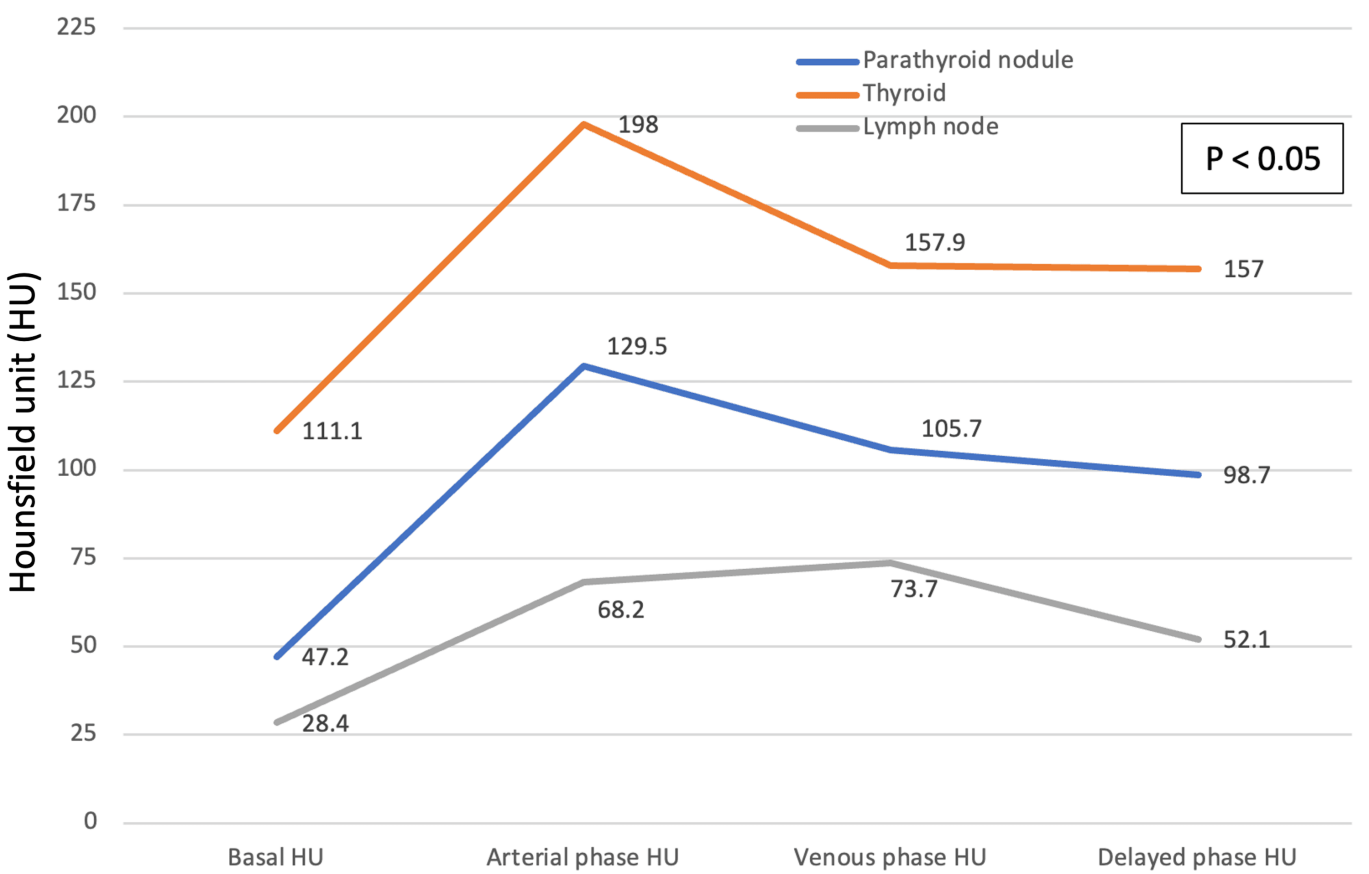
Dynamic Contrast Enhancement Curve of the Parathyroid Adenoma, Thyroid, and Lymph Node. Parathyroid adenomas exhibited low baseline attenuation, marked arterial-phase enhancement, and venous wash-out. Thyroid tissue showed higher baseline HU, moderate arterial enhancement, and persistent venous-phase enhancement. Lymph nodes demonstrated low baseline HU with progressive enhancement peaking in the venous phase. Differences between parathyroid lesions and both thyroid and lymph nodes across phases were statistically significant (p<0.05).

**Figure 3:**
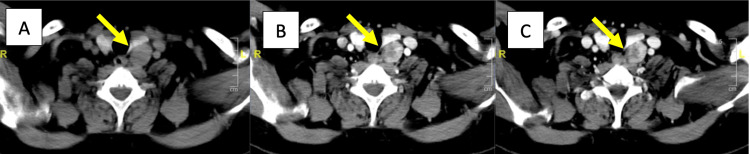
4D CT Scan for Parathyroid Lesion in a 46-Year-Old Man. 4D CT scan depicting a left inferior parathyroid adenoma measuring 3.6×2.1×2.1 cm, visualized in the plain (A), arterial (B), and venous (C) phases. The lesion demonstrates attenuation values of 48 HU, 112 HU, and 72 HU, respectively, across these phases. The percentage arterial enhancement (PAE) of the lesion was calculated to be 147.6%. 4D CT, four-dimensional computed tomography; HU, Hounsfield units

CT attenuation characteristics of parathyroid lesions were distinct compared to thyroid parenchyma and lymph nodes. Parathyroid lesions showed significantly lower attenuation compared to the thyroid and higher enhancement than lymph nodes at baseline and during arterial and venous phases, respectively (Figure 2). The peak arterial enhancement of parathyroid lesions, thyroid parenchyma, and lymph nodes was 129.5 HU, 198 HU, and 68.2 HU, respectively. The PAE of parathyroid lesions was 185.7 (IQR: 109.7-248.6), which was significantly higher than that of thyroid tissue (62.7 {41.6-108.2}, p<0.05) but not significantly different from that of lymph nodes (111.5 {70.6-219.7}, p=0.46). The application of the previously published PAE cutoff of 128.9% (as proposed by Goroshi et al. [13]) performed suboptimally in our cohort. Using this threshold, the sensitivity was 75%, and the specificity was 31.6%, with a poor area under the curve (0.386), indicating limited diagnostic utility in our population. A representative 4D CT scan image of a parathyroid adenoma in a 46-year-old man with PHPT is shown in Figure 3. The lesion demonstrated attenuation values of 48 HU (plain phase), 112 HU (arterial phase), and 72 HU (venous phase). In comparison, the corresponding values for thyroid tissue were 109 HU, 188 HU, and 193 HU, while for a lymph node, they were 15 HU, 51 HU, and 63 HU, respectively. The PAE of the parathyroid lesion was 147.6%, compared to 72.5% for the thyroid.

## Discussion

In this retrospective analysis of PHPT cases managed at our center over the past decade, we identified 53 patients who underwent preoperative localization using all three imaging modalities, USG, 99mTc-sestamibi SPECT/CT, and 4D CT, at our institution. We are currently routinely doing all investigations to develop a protocol. The mean age of the cohort was 42.7±14.7 years, with a slight female predominance (54.7%). Median baseline iPTH levels were 328 pg/mL. The demographic characteristics of our cohort are largely consistent with data from the Indian PHPT registry [15]. The majority of patients had typical parathyroid adenomas (n=49), while three cases were confirmed as carcinoma and one as an atypical adenoma. Lesions were most frequently located in the right inferior gland (47%), followed by the left inferior (31%). Overall, preoperative imaging successfully localized the pathological gland in 94.34% of cases. This is a promising finding, particularly considering that a prior Indian study utilizing advanced modalities such as 4D magnetic resonance imaging (MRI) and 4D CT reported a comparable localization rate of 96.7% [16].

In our study, 4D CT demonstrated greater sensitivity compared to USG and 99mTc-sestamibi SPECT/CT, particularly in detecting smaller adenomas. For lesions of less than 2 cm, 4D CT achieved a sensitivity of 100%, outperforming USG (76.2%) and sestamibi (71.4%). These findings reinforce the utility of 4D CT as a highly sensitive imaging modality in the preoperative evaluation of PHPT. Two meta-analyses evaluating these three imaging modalities corroborate our findings [17,18] and are largely consistent with studies across India [19]. While all three modalities exhibited high PPVs, the inter-modality agreement was notably low, highlighting the complementary role of 4D CT, especially in cases with inconclusive or discordant findings on USG or sestamibi. Notably, 4D CT successfully localized 20 lesions that were either missed or mislocalized by USG or 99mTc-sestamibi SPECT/CT.

Cervical lymph nodes and thyroid nodules are well-recognized mimickers of parathyroid adenomas. To address this diagnostic challenge, we analyzed their dynamic enhancement patterns on 4D CT. We observed significant differences in attenuation across baseline, arterial, and venous phases among parathyroid, thyroid, and lymph node lesions. Parathyroid adenomas demonstrated characteristic arterial-phase hyperenhancement. In contrast, thyroid tissue showed greater baseline enhancement, lower percentage arterial enhancement (PAE) compared to parathyroid lesions, and persistent enhancement in the venous phase. Lymph nodes displayed distinctly different dynamics, with progressive enhancement mainly during the venous phase. Given that lymph nodes generally lack arterial-phase enhancement, as supported by existing literature, calculating PAE for differentiating lymph nodes from parathyroid lesions may be misleading and is not recommended [20].

An important objective of our study was to evaluate the diagnostic utility of percentage arterial enhancement in localizing parathyroid adenomas. The diagnostic performance of the previously published PAE threshold of 128.9%, proposed by Goroshi et al., did not replicate effectively in our cohort [13]. In our analysis, 25.8% of biochemically and surgically confirmed parathyroid adenomas did not exceed this threshold. It is noteworthy that a considerable proportion of parathyroid adenomas do not exhibit distinct enhancement during the arterial phase. These are categorized as type B (showing enhancement predominantly in the delayed phase) and type C (appearing more distinct on the non-contrast phase) [21]. These type B and C parathyroid adenomas are known to demonstrate relatively lower enhancement [22]. While Goroshi et al. reported excellent sensitivity and specificity using this cutoff, we observed a sensitivity of 75% and a specificity of only 31.6% [13]. This discrepancy may be partially attributed to variations in arterial-phase acquisition protocols across institutions. For instance, Murugan et al. [16] performed arterial-phase imaging at 25 seconds post-contrast injection, whereas we acquired arterial-phase images at 10-15 seconds and Goroshi et al. [13] at 20 seconds. These differences in timing, along with potential differences in scanner parameters and lesion vascularity, may influence enhancement dynamics. Therefore, despite being validated in a prospective cohort, the fixed cutoff value may not be universally applicable across different imaging protocols and populations [23].

Debnam et al., in their study distinguishing intrathyroidal parathyroid adenomas from colloid nodules and papillary thyroid carcinoma, reported that wash-in percentages (PAE) did not significantly differ among the lesions [24]. Instead, they emphasized rapid wash-out as a more distinguishing feature of intrathyroid parathyroid adenomas. In contrast, Vu et al. demonstrated that the PAE was the most powerful discriminator, with contrast wash-out from the arterial to venous phase being less effective [25]. They observed that parathyroid adenomas tend to be hypodense relative to thyroid tissue and exhibit avid early contrast enhancement, with significantly higher PAE values (493% versus 132% for thyroid, P<0.05). Raeymaeckers et al. further noted that the slope of enhancement increase was significantly steeper and the time to peak significantly earlier in parathyroid adenomas compared to normal thyroid tissue. However, none of these studies established a specific diagnostic cutoff [26].

This study has some limitations. First, its retrospective design may introduce selection bias, and certain data points, such as exact contrast timing protocols or complete histopathological confirmation, were not uniformly available across all cases. Second, we did not assess inter-observer agreement between the two radiologists, which could have contributed to diagnostic variability. The lack of blinding of radiologists to clinical and other imaging findings may also introduce bias in image interpretation. The USG was performed by different radiologists as part of routine clinical evaluation and not in a blinded fashion. Finally, although our study included a reasonably sized cohort, larger prospective multicentric studies are needed to validate the optimal PAE threshold and to refine imaging protocols tailored for the Indian population. 18F-Fluorocholine PET/CT shows promise as a sensitive imaging tool for parathyroid adenomas, with good concordance with 4D CT. However, current evidence is limited, and its routine clinical use awaits further validation.

## Conclusions

In this retrospective study, 4D CT showed higher sensitivity than USG and 99mTc-sestamibi SPECT/CT for localizing parathyroid adenomas. It adds diagnostic value in cases with discordant or negative findings on routine modalities. While dynamic enhancement patterns helped distinguish adenomas from mimics, the application of fixed arterial enhancement thresholds was limited. The retrospective nature of the study and protocol variability remain important limitations and should be considered when interpreting these findings.

## Appendices

Data collection proforma

Project title: Diagnostic accuracy of four-dimensional computed tomography (4D CT) scan in detecting parathyroid adenoma compared with ultrasound and technetium sestamibi SPECT/CT in primary hyperparathyroidism

Section A: Patient Demographics

Study ID: _______

Age: _______

Gender: Male/Female

Section B: Biochemical Parameters

Serum Calcium (mg/dL): _______

Serum Phosphorus (mg/dL): _______

Parathyroid Hormone (PTH) Level (pg/mL): _______

Vitamin D Level (ng/mL): _______

Alkaline Phosphatase (IU/L): _______

Section C: Imaging Modalities

Ultrasound findings:

Parathyroid Lesion: Yes/No

Size of Lesion (mm): _______

Location (Right/Left and Superior/Inferior): _______

Other Notes: _______

Technetium sestamibi SPECT/CT findings:

Parathyroid Lesion: Yes/No

Size of Lesion (mm): _______

Location (Right/Left and Superior/Inferior): _______

Other Notes: _______

4D CT scan findings:

Parathyroid Lesion: Yes/No

Location (Right/Left and Superior/Inferior): _______

Size (mm): _______

Peak Arterial Enhancement Percentage: _______

Wash-In Characteristics: _______

Wash-Out Characteristics: _______

Other Notes: _______

Section D: Diagnostic Comparison

Preoperative localization agreement:

Concordance Between Ultrasound and 4D CT: Yes/No

Concordance Between Sestamibi SPECT/CT and 4D CT: Yes/No

Surgical findings:

Confirmed Parathyroid Adenoma: Yes/No

Location (Right/Left and Superior/Inferior): _______

Size of Adenoma (mm): _______

Section E: Radiological Features Comparison

Comparison with thyroid adenoma:

Size (mm): _______

Enhancement Pattern (Peak Arterial Enhancement, Wash-In, and Wash-Out): _______

Other Features: _______

Comparison with lymph nodes:

Size (mm): _______

Enhancement Pattern (Peak Arterial Enhancement, Wash-In, and Wash-Out): _______
